# Natural honey acts as a nonpermeating cryoprotectant for promoting bovine oocyte vitrification

**DOI:** 10.1371/journal.pone.0238573

**Published:** 2020-09-02

**Authors:** Bilal Alfoteisy, Jaswant Singh, Muhammad Anzar

**Affiliations:** 1 Department of Veterinary Biomedical Sciences, Western College of Veterinary Medicine, University of Saskatchewan, Saskatoon, SK, Canada; 2 Agriculture and Agri-Food Canada, Saskatoon Research and Development Center, Canadian Animal Genetic Resource Program, Saskatoon, SK, Canada; University of Connecticut, UNITED STATES

## Abstract

Sugars are commonly supplemented into vitrification solution to dehydrate cells in order to reduce the formation of fatal intracellular ice crystals. Natural honey is a mixture of 25 sugars (mainly fructose and glucose) that have different biological and pharmacological benefits. The present study was designed to determine if honey can be used as a nonpermeating cryoprotectant in vitrification of bovine oocytes. In the first experiment, denuded-MII oocytes were exposed to 0.25, 0.5, 1.0, 1.5 or 2.0 M of honey or sucrose. Natural honey and sucrose caused similar ooplasm dehydration. A significant relationship existed between time and ooplasm volume change (P < 0.05), during dehydration and rehydration phases, in both honey and sucrose solutions. In the second experiment, the immature cumulus-oocyte complexes (COCs) were vitrified in an EG/DMSO-based vitrification solution containing honey (0.5, 1 or 1.5 M) or sucrose (0.5 M) as a gold standard. The vitrified-warmed COCs were matured *in vitro* and evaluated for nuclear maturation. The maturation (MII) rate was greater in nonvitrified control (81%) than vitrified groups (54%, P < 0.05). In the third experiment, COCs were either remained nonvitrified (control) or vitrified in 1.0 M honey or 0.5 M sucrose, followed by IVM, IVF and IVC (for 9 days). Cleavage rate was greater in control (74%) than in vitrified groups (47%, P < 0.05), without significant difference between sugars. Blastocyst rate was 34, 13 and 3% in control, honey and sucrose groups respectively (P < 0.05). In conclusion, natural honey acted as a nonpermeating cryoprotectant in vitrification solution and improved the embryonic development in vitrified bovine COCs.

## Introduction

Sugars are commonly used as energy substrate and/or nonpermeating cryoprotectants (CPs) in cryoprotective solution. Sugars, being nonpermeating CPs, cause an osmotic gradient across the cell membrane that enhances cell dehydration before freezing to reduce the quantity of intracellular water. This reduction in water quantity decreases intracellular ice formation and thus minimizes lethal freezing injuries [1, 2]. Sugars enhance the viscosity of the intracellular compartment, decrease the concentration of permeating CPs and thus lessen the associated intracellular toxicity [3, 4]. Furthermore, vitrification solution containing sugars improved the survival of bovine blastocysts and human immature oocytes following vitrification [5, 6].

Monosaccharides such as fructose and glucose are used for semen cryopreservation without any cytotoxic effect [7, 8]. They can be mixed easily and efficiently even in concentrated solutions of CPs because they have a lower viscosity than disaccharides [9]. Fructose has a better effect on semen quality than disaccharides and oligosaccharides for cryopreservation of red deer sperm [10]. Interestingly, disaccharides like sucrose and trehalose (not lactose) increased sperm viability and reduced damage to acrosome in dog sperm whereas fructose improved both intact acrosome and motility [11]. Other researchers showed that the effect of different sugars during mouse sperm cryopreservation depends on their mass concentrations instead of their molar concentrations [12]. Sucrose, trehalose [13] and lactose [14] have been used as nonpermeating CPs in vitrification solutions, but sucrose and trehalose are more common than other sugars [15]. Raffinose (trisaccharide) has also proven to be effective in increasing the survival rate of embryos after vitrification [15]. Furthermore, the mixture of two sugars (sucrose and glucose) in vitrification solution improved the survival rate of the vitrified bovine blastocysts more than the addition of sucrose alone [6].

Natural honey is a mixture of 25 sugars (mainly fructose and glucose) accounting for approximately 95% of its dry matter [16–18]. Besides its dominant components of saccharides, traces of a large number of other bioactive substances such as organic acids, enzymes, antioxidants and vitamins are present in honey. Such a unique composition provides numerous nutritional, biological and pharmacological effects on living cells, *i*.*e*. antimicrobial (antiviral, antibacterial and antifungal), antioxidant and antitoxin, anti-inflammatory, antimutagenic, anticancer and immunosuppressive activities [16–22].

Cryopreservation of oocytes has been very challenging due to their unique structure and sensitivity to chilling [23]. Several attempts were made to improve the blastocyst rate following vitrification of germinal vesicle (GV)-stage bovine oocytes. Cholesterol-loaded cyclodextrin improved the survival of bovine oocytes [24] but could not improve the blastocyst development [25]. Similarly, the pretreatment of GV oocytes with cytoskeletal relaxant cytochalasin B did not improve the blastocyst rate of vitrified GV oocytes [26]. Recently, blastocyst formation tended to improve by maturing the vitrified-warmed immature oocytes in the presence of cyclic adenosine monophosphate modulators forskolin or 3-isobutyl-1-methylxanthine (IBMX) [27]. There are conflicting reports on the role of cumulus cells in the development of GV oocytes following vitrification and warming. Earlier, the cumulus-enclosed GV oocytes showed higher cleavage and blastocyst rates than partially denuded GV oocytes [28]. Recently, the downsizing of cumulus cell layers surrounding GV oocyte improved the blastocyst rate up to 30% [29]. To the best of our knowledge, natural honey has not yet been studied as a nonpermeating CP for vitrification of oocytes. Since honey is a rich-mixture of sugars, it was hypothesized that natural (unheated) honey in vitrification solution can induce sufficient cell dehydration in bovine oocytes and adds biological benefits towards early embryonic development.

The overall objective of this study was to investigate if natural honey can be used as a nonpermeating CP for vitrification of bovine oocytes and subsequent early embryonic development. The specific objectives of this study were to determine osmolalities of different concentrations of honey and sucrose, to compare the volumetric change in bovine oocytes in different concentrations of natural honey and sucrose solutions, and to investigate *in vitro* maturation (IVM), fertilization (IVF) and embryo development (IVC) of GV-stage oocytes vitrified in honey and sucrose solutions.

## Materials and methods

The biological procedures used in this study were approved by the University Committee on Animal Care and Supply, Animal Research Ethics Board, University of Saskatchewan (Animal Use Protocol # 20090155).

### Chemicals and supplies

Dulbecco’s phosphate buffered saline (DPBS), Ca^2+^/Mg^2+^-free DPBS, newborn calf serum (CS), tissue culture medium (TCM)-199 and minimum essential medium (MEM) non-essential amino acids were purchased from Invitrogen Inc. (Burlington, ON, Canada). Lutropin-V (LH) and folltropin-V (FSH) were supplied by Bioniche Animal Health, Inc. (Belleville, ON, Canada). All other chemicals and reagents were purchased from Sigma-Aldrich (Oakville, ON, Canada), unless otherwise stated.

### Preparation of natural honey or sucrose containing solutions

Natural honey (unprocessed) was procured from a local beekeeper (Sollosy’s Honey, T&H Apiaries, Saskatoon, Canada). First, the osmotic pressure of 10% w/v natural honey solution (10% w/v in water) was determined using a vapor-pressure osmometer (VAPOR^®^, model # 5520, Wescor Inc. Logan, Utah, USA). It was calculated that 21.74 gm honey in 100 ml water can yield an osmotic pressure of 1000 ± 10 mOsm/kg and thus was considered equivalent to 1 M. For confirmation, honey and sucrose solutions of 0.25, 0.5 and 1.0 M were prepared and relationships between molal concentrations and osmolalities were determined. Later, various concentrations of honey and sucrose ranging from 0.25 to 2 M were used, for vitrification of oocytes, in experiments on IVM, IVF and IVC.

### Oocyte collection

Bovine ovaries were collected from cows slaughtered under strict regulations of the Canadian Food Inspection Agency, Government of Canada. Ovaries were transported to the laboratory at approximately 25°C within 3 h, and initially processed as previously described [30]. Briefly, ovaries were washed in 0.15 M NaCl and extra-ovarian tissues were removed. The immature cumulus-oocytes complexes (COCs) GV-stage were aspirated from follicles (3–8 mm in diameter) using an 18-gauge needle attached to a 5-ml syringe containing 1-ml of DPBS supplemented with 5% new-born calf serum (CS, Invitrogen Inc.; v/v). The follicular fluid aspirated from different ovaries was pooled, and COCs were searched under a stereomicroscope (10 × magnification) and evaluated according to the International Embryo Transfer Society guidelines.

### Experiment 1: Dehydration of bovine oocytes (MII-stage) in honey and sucrose solutions

#### IVM

Bovine oocytes with uniform cytoplasm and 3–4 layers of cumulus cells (Grade 1) were selected and washed (3 ×) in maturation media [TCM-199 supplemented with 5% CS (collectively called as “TCM-CS”), 0.5 μg/ml FSH, 5 μg/ml LH and 50 μg/ml gentamicin]. Groups of 18 to 22 COCs were incubated in 100-μl droplets of maturation media (under mineral oil) at 38.5°C, 5% CO_2_, and high humidity in air for 22 h. After IVM, COCs were denuded with 0.3% hyaluronidase in Ca^2+^/Mg^2+^-free DPBS and only matured oocytes (MII-stage) possessing the first polar body with homogeneous ooplasm were selected under stereomicroscope to study dehydration and rehydration phases, as described below.

#### Image processing of bovine COCs during dehydration and rehydration phases

First, five concentrations (0.25, 0.5, 1, 1.5 and 2 M) of honey and sucrose were prepared. Honey concentrations were achieved by dissolving 5.4, 10.9, 21.7, 32.6 and 43.5 gm of honey per 100 ml of TCM-CS. Denuded oocytes (MII-stage) were incubated in TCM-CS at 38.5°C in a 5% CO_2_ air and high humidity for at least 3 h before undergoing dehydration and rehydration phases. Oocytes were randomly distributed into 11 groups, *i*.*e*. control (no sugar) and five concentrations of each honey or sucrose group (0.25, 0.5, 1.0, 1.5 or 2.0 M in TCM-CS). For imaging, a mature oocyte was held with an ICSI micropipette (MIC-50-30, Humagen, Jyllinge, Denmark) under an inverted Nikon microscope equipped with micromanipulators (TransferMan NK_2_, Eppendrof AG 2231, Hamburg, Germany) and a Nikon D90 camera. Each oocyte was first equilibrated in 20-μl and then 40-μl droplets of the control solution (TCM-CS) for 10 s and 1 min, respectively. After equilibration, oocyte was transferred into 40-μl droplet of an experimental solution (TCM-CS + honey or sucrose) for 3 min, for imaging during dehydration. Afterwards, oocyte was transferred into 40-μl droplet of control solution (TCM-CS) for 3 min, for imaging during rehydration. During dehydration or rehydration phases, the images were captured from recorded videos at 0, 5, 10, 15, 20, 25, 30, 60, 90, 120, 150 and 180 s using VideoMach software (version 5.8.3; www.gromada.com) running under Microsoft Windows 7 Professional. A total of 24 images were captured from each oocyte and these images were overlaid to evaluate volumetric changes during the dehydration and rehydration phases. Image-J version 1.42 software (Wayne Rasband, National Institute of Mental Health, Bethesda, Maryland, USA) was used to measure the minimum and maximum diameters of ooplasm. The volume of ooplasm was calculated using the following formula, assuming oocyte as a sphere:

Ooplasmvolume=4/3*π*r1*r2*r3

where r1 and r2 are maximum and minimum radii, and r3 was assumed to be equal to the minimum radius.

The shrinkage of ooplasm at a given concentration of honey or sucrose was calculated as follows:

OoplasmvolumeinCPsolution/OoplasmvolumeincontrolTCMwithoutCP*100

### Experiment 2: IVM of bovine oocytes (GV-stage) vitrified in honey and sucrose solutions

This experiment was designed to determine the post-warm IVM ability of bovine oocytes (GV-stage) vitrified in a solution containing honey or sucrose as a nonpermeating CP. After collection and washing, COCs were distributed randomly into five groups as follows: control (no vitrification), 0.5 M sucrose (gold standard), 0.5 M honey, 1.0 M honey and 1.5 M honey. COCs were vitrified, warmed, cultured *in vitro* and evaluated for nuclear maturation as described below.

#### Vitrification and warming procedures

Vitrification of COCs (GV-stage) was performed using the Cryotop method [30, 31] with some modifications. All COCs were first equilibrated in vitrification solution 1 [(VS1; TCM-199 + 7.5% v/v dimethyl sulfoxide (DMSO) + 7.5% v/v ethylene glycol (EG) + 20% v/v CS] at 22°C for 5 min. After equilibration, COCs were transferred through three 30-μl droplets of vitrification solution 2 (VS2) which contained either sucrose (0.5 M) or natural honey (1 M) in TCM-199 + 15% v/v EG + 15% v/v DMSO + 20% v/v CS at 37°C for 1 min. Then, 3–5 COCs were loaded onto the filmstrip of a Cryotop (Kitazato Corp.; Shizuoka, Japan) and immediately plunged into liquid nitrogen. Prior to vitrification, the surrounding media was removed by gentle aspiration. For warming, the Cryotop containing COCs was immersed in 38.5°C warming solution consisting of TCM-199 + 20% v/v CS supplemented with the desired concentration of natural honey (0.5, 1, 1.5 M) or sucrose (0.5 M) for 1 min. After warming, COCs were washed (3 ×, 5 min each) in TCM-CS.

#### Assessment of nuclear maturation (lamin-AC/DAPI staining)

The vitrified-thawed COCs were washed (3 ×) in the maturation media mentioned in Experiment 1. The COCs (n = 18 to 20) were placed in 100-μl IVM media droplets under mineral oil, and incubated at 38.5°C, 5% CO_2_ in air and high humidity, for 22 h.

The nuclear maturation of oocytes was determined using lamin-AC/DAPI staining, as previously described [32]. After IVM, the vitrified-warmed COCs were completely denuded (by pipetting 80 to 100 times avoiding bubble formation) in 0.3% hyaluronidase in Ca^2+^/Mg^2+^-free DPBS. Denuded oocytes were fixed in 4% w/v paraformaldehyde in DPBS for 15 min. All subsequent steps were performed at 22°C and oocytes were washed (3 ×, 5 min each) in DPBS after each step. Oocytes were permeabilized with 0.5% v/v Triton X-100 in DPBS for 30 min followed by an additional 30 min permeabilization in 0.05% v/v Tween-20 (BIO-RAD, Hercules, CA, USA) in DBPS. Afterwards, oocytes were transferred in a blocking buffer [2% w/v bovine serum albumin (BSA) in DPBS] at 22°C for 60 min or at 4°C overnight. After blocking, oocytes were incubated with mouse anti-lamin-AC (1:300; Santa Cruz Biotechnology, Santa Cruz, CA, USA) in DPBS + 2% w/v BSA for 60 min followed by washing (3 ×, 5 min each) and incubation in secondary antibody, Alexa 488 labelled anti-mouse IgG (Santa Cruz Biotechnology) in 2% w/v BSA in DPBS (1:200), for 60 min. Oocytes were washed (3 ×, 5 min each) and transferred through at least three 5-μl droplets of Vectashield Mounting Medium containing 1.5 μg/ml of DAPI (Vector Laboratories Inc., Burlingame, CA 94010 USA). Oocytes were mounted on a glass slide under a coverslip supported with paraffin-vaseline (1:1) drops at each corner of the coverslip to avoid oocyte rupture. Finally, oocytes were evaluated for nuclear maturation and classified as GV, germinal vesicle breakdown (GVBD), metaphase I (MI), and MII as previously described [32]. Oocytes reaching MII-stage were included for statistical analysis.

### Experiment 3: IVF and IVC of bovine oocytes (GV-stage) vitrified in honey and sucrose solutions

This experiment was designed to investigate cleavage and blastocyst rates of bovine oocytes (GV-stage) vitrified in a solution containing 0.5 M sucrose as a gold standard (17.1% w/v) or 1.0 M honey (21.7% w/v). Briefly, COCs were randomly distributed into three groups including nonvitrified (control) and vitrified groups using honey or sucrose, and vitrified as described above (Experiment 2).

#### IVF and IVC

Frozen semen from a bull was used for all treatments and replicates. Thawed semen was washed through Percoll gradient (45 and 90%) [33]. After washing, sperm were diluted in Brackett-Oliphant (BO) medium [34] to a concentration of 3 × 10^6^ cells/ml. COCs (n = 18–22) were washed (3 ×) in BO medium supplemented with 10% w/v BSA and then placed into 100-μl droplets of sperm suspension in BO medium, under mineral oil, for co-incubation at 38.5°C, 5% CO_2_ in air and high humidity, for 18 h. After co-incubation, the presumptive zygotes were denuded and washed (3 ×) through IVC medium, *i*.*e*. Charles-Rosenkrans amino acid (CR1aa) with 5% v/v CS, 1% v/v MEM non-essential amino acids, 2% v/v BME essential amino acids (Invitrogen Inc.), 1% v/v L-Glutamic acid, 0.3% w/v BSA and 0.05 μg/ml gentamicin. Finally, 18–22 presumptive zygotes were transferred in 100-μl droplets of IVC medium under mineral oil and incubated at 38.5°C under 5% CO_2_, 5% O_2_, 90% N_2_ and high humidity. During IVC, the cleavage rate was determined at day 2 of *IVF*; whereas, the blastocyst formation rate was evaluated at day 9.

### Statistical analysis

Data were presented as means ± SEM. Data were analyzed using SAS^®^ Enterprise Guide 4.2 (SAS, SAS Enterprise Guide Inc., 4.2 ed. Cary, NC, USA; 2006). The statistical analysis included following: (a) Proc Mixed repeated-measures one-way analysis of variance (time as independent discrete variable with randomized complete block design) in order to determine the time point by which the dehydration or rehydration is completed (*i*.*e*. equilibrium is obtained) in each concentration of honey- and sucrose-based solutions. (b) Proc Mixed repeated-measures factorial (compound*concentration*time) analysis of variance (time as independent discrete variable) with randomized complete block design was carried out in order to determine the effect of CP, concentration and time on ooplasm volume during dehydration and rehydration. Regression analysis was used to study the relationships of molal concentrations of sucrose and honey with osmolalities, and ooplasm shrinkage at 60 s during dehydration. Polynomial regression analysis was used to study the curvilinear relationship between time and changes in ooplasm volume, during dehydration and rehydration phases, in different concentrations of honey and sucrose.

In the second experiment, differences in oocyte maturation rate among treatment groups were analyzed using one-way analysis of variance. In the third experiment, ooplasm shrinkage, at 60 s during dehydration, in 0.5 M sucrose and 1 M honey was compared with t-test. The statistical analyses of cleavage and blastocyst formation rates were carried out using chi-square (Fisher's exact test for the blastocyst formation rates). The differences between treatment means were considered significant at a level of P < 0.05.

## Results

### Experiment 1: Dehydration of bovine oocytes (MII-stage) in honey and sucrose solutions

**Fig 1:**
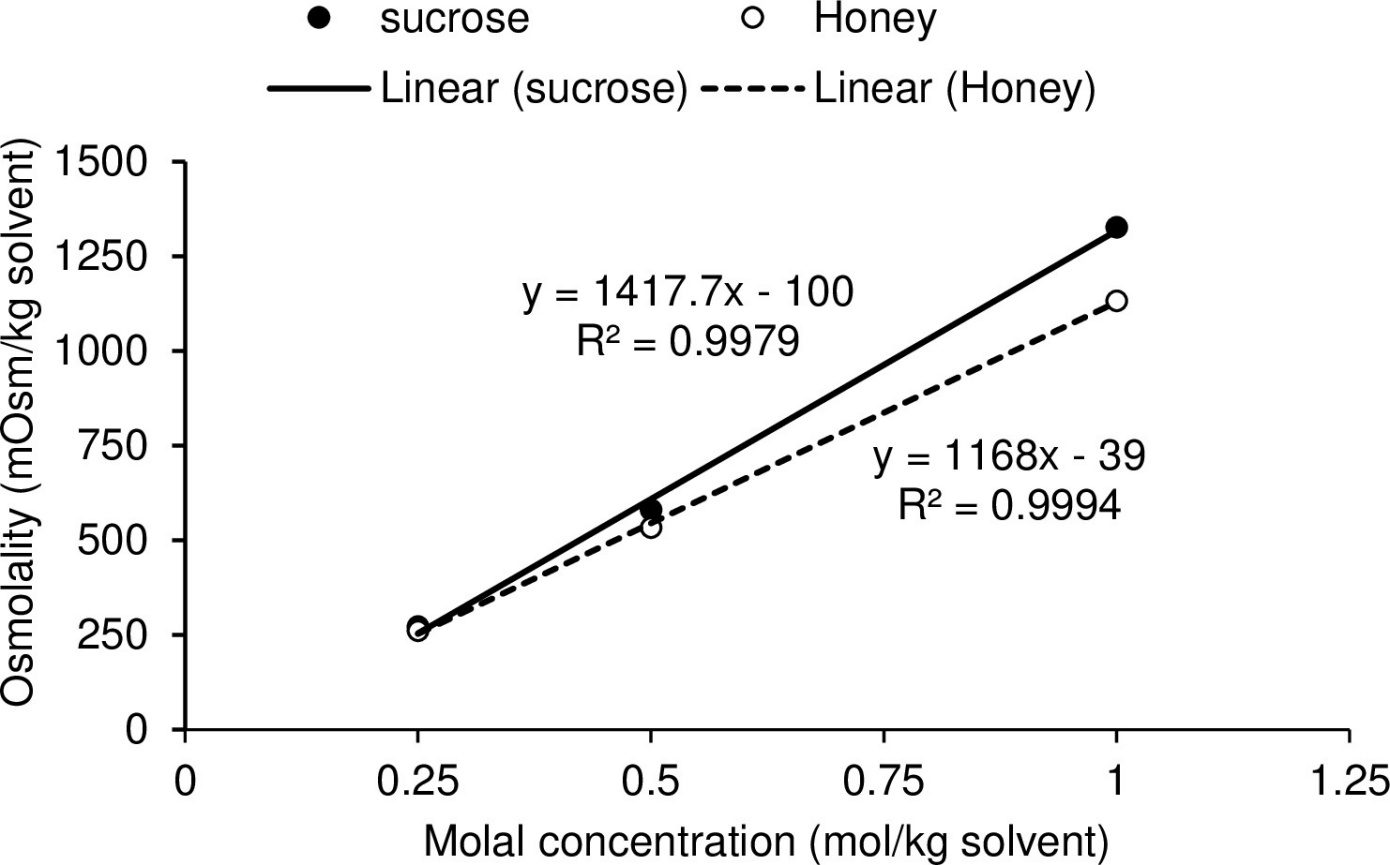
Relationship between molal concentrations and osmolalities of 0.25, 0.5 and 1 M honey and sucrose solutions.

**Fig 2:**
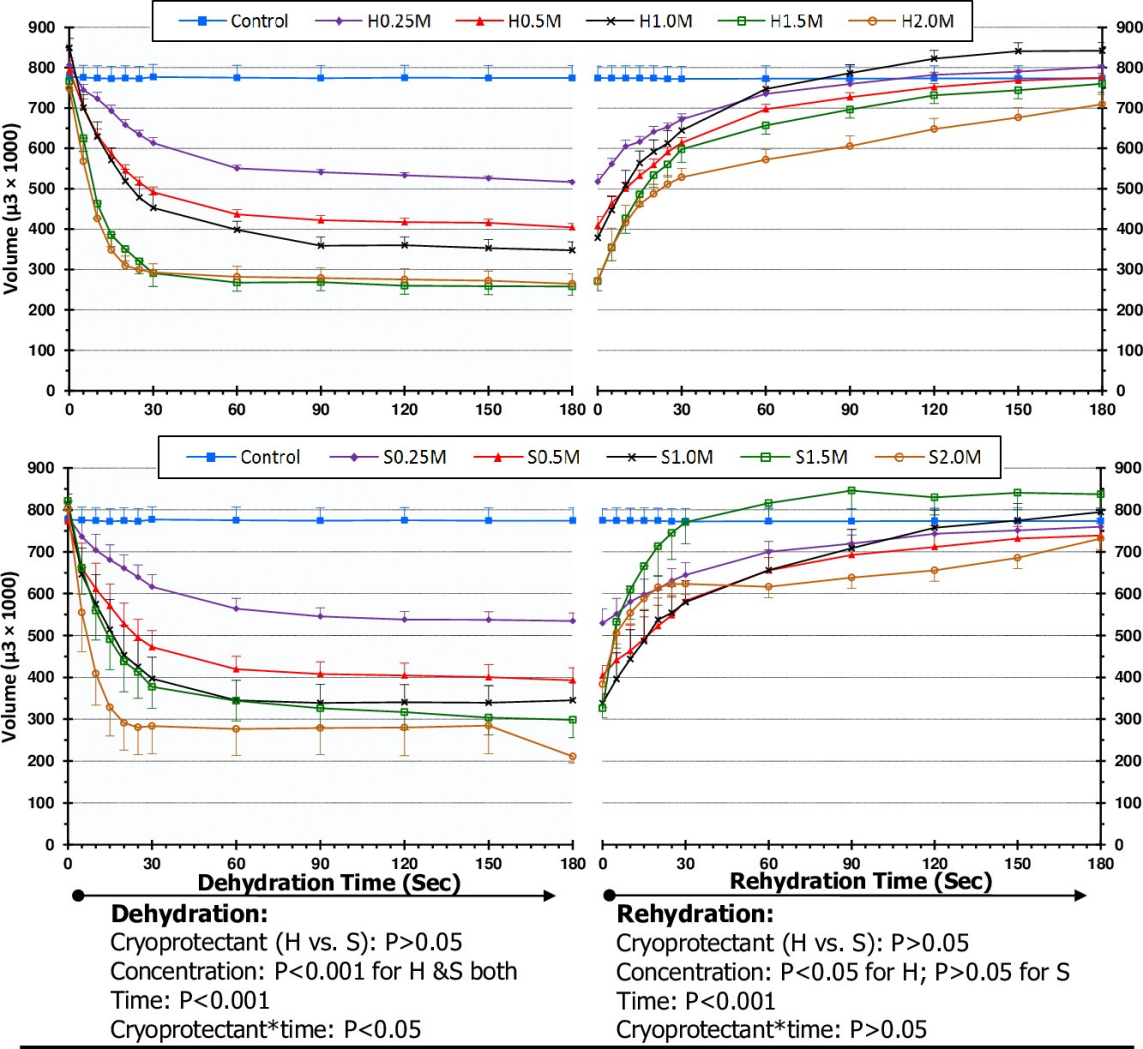
Effect of honey (H) and sucrose (S) concentrations (0.25, 0.5, 1, 1.5 and 2 M), time (0, 5, 10, 15, 20, 25, 30, 60, 90, 120, 150 and 180 s) and their interactions on ooplasm volume (μ^3^ × 1000) during dehydration and rehydration phases. Each point represents a mean ± SEM (n = 3 oocytes per treatment group per replicate × 3 replicates).

**Fig 3:**
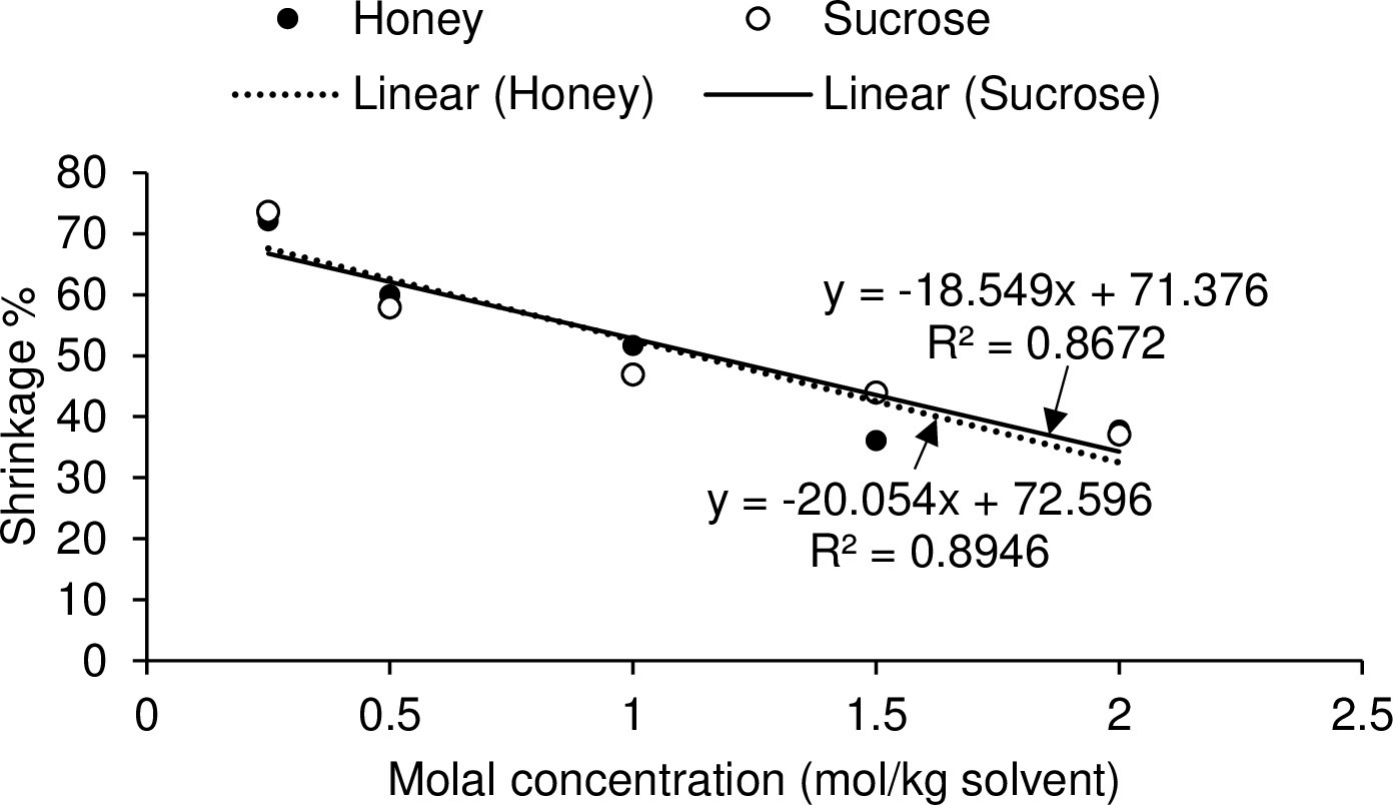
Relationship between molal concentrations of honey and sucrose, and ooplasm shrinkage at 60 s during dehydration phase (n = 3 oocytes per treatment group per replicate × 3 replicates).

Linear relationships existed between molal concentrations and osmolalities in both honey and sucrose solutions (Fig 1). Total volume of bovine oocytes (including zona pellucida) remained the same during dehydration and rehydration processes. No significant difference was observed in ooplasm volume and shrinkage due to nonpermeating CPs (honey and sucrose) during dehydration (P = 0.485) and rehydration (P = 0.990) phases (Fig 2). In this study, the time required for maximum dehydration in bovine oocytes was 60 s in all concentrations of honey and sucrose (Fig 2). During dehydration phase, the ooplasm volume decreased (P < 0.05) and shrinkage relative to control solution increased (P < 0.05) with an increase in concentrations of honey and sucrose, at 60 s (Table 1). At 60 s, ooplasm shrinkage ranged from 72 to 38% and 74 to 37% in different concentrations of honey and sucrose, respectively. First significant shrinkage in ooplasm was observed in 1.5 M honey and 1 M sucrose (Table 1). There were significant linear relationships between molal concentrations of honey and sucrose, and ooplasm shrinkage at 60 s during dehydration (Fig 3). During rehydration in the isosmotic TCM-CS solution, ooplasm regained their original volume in 60 s (Fig 2).

**Table 1:** Volume of bovine ooplasm in TCM containing different concentrations of honey and sucrose at 60 s post-exposure, and their shrinkage (%) relative to volume in the control TCM (without nonpermeating CP). Each value represents mean ± SEM (n = 9 oocytes).

Conc.(Mol/L)	Honey*		Sucrose*	
	Volume (μ^3^ × 1000)	Shrinkage (%)	Volume (μ^3^ × 1000)	Shrinkage (%)
0	772 ± 31.2^a^	NA	772 ± 31.2^a^	NA
0.25	550 ± 8.2^b^	72 ± 2.4^a^	564 ± 24.6^b^	74 ± 4.2^a^
0.50	436 ± 12.3^b^	60 ± 2.3^a^	419 ± 23.7^b^	58 ± 4.1^a^
1.00	398 ± 34.1^b^	52 ± 3.7^a^^b^	345 ± 30.2^c^	47 ± 5.1^b^
1.50	268 ± 21.8^c^	36 ± 3.7^b^	343 ± 47.9^c^	44 ± 5.6^b^
2.00	277 ± 63.5^c^	38 ± 4.2^b^	282 ± 25.7^c^	37 ± 9.7^b^

*At a given concentration, the ooplasm volume and shrinkage between honey and sucrose were similar (P > 0.05).

^a,b^Within a column, values with different superscripts differ (P < 0.05).

NA–Not applicable.

Polynomial regression revealed that, the ooplasm shrinkage during dehydration and swelling during rehydration in all honey and sucrose concentrations changed with time (P < 0.05, Table 2).

**Table 2:** Polynomial regression analysis of ooplasm volume (y) as a function of time (x), during dehydration and rehydration phases, in different concentrations of nonpermeating cryoprotectants (NPCP; honey and sucrose).

Phase/NPCP	Conc. (Mol/L) (Mol/L)	Polynomial regression	Correlation coefficient
Dehydration			
Control	0	y = 0.00002x^2^–0.0051x +764.8	r = 0.104, P > 0.05
Sucrose	0.25	y = -0.0002x^3^ + 0.0553x^2^–6.3255x +768.38	r = -0.995, P < 0.01
Honey	0.25	y = -0.0002x^3^ + 0.0687x^2^–7.5136x + 789.79	r = -0.995, P < 0.01
Sucrose	0.5	y = -0.0003x^3^ + 0.1088x^2^–10.951x +723.66	r = -0.980, P < 0.01
Honey	0.5	y = -0.0003x^3^ + 0.1128x^2^–11.365x + 751.48	r = -0.982, P < 0.01
Sucrose	1.0	y = -0.0005x^3^ + 0.1532x^2^–14.842x + 730.65	r = -0.962, P < 0.01
Honey	1.0	y = -0.0004x^3^ + 0.1396x^2^–14.141x + 777.82	r = -0.975, P < 0.01
Sucrose	1.5	y = -0.0005x^3^ + 0.1611x^2^–15.481x + 730.95	r = -0.956, P < 0.01
Honey	1.5	y = -0.0006x^3^ + 0.1839x^2^–16.999x + 669.17	r = -0.934, P < 0.01
Sucrose	2.0	y = -0.0007x^3^ + 0.2135x^2^–18.139x + 643.46	r = -0.886, P < 0.01
Honey	2.0	y = -0.0006x^3^ + 0.178x^2^–15.868x + 624.84	r = -0.899, P < 0.01
Rehydration			
Control	0	y = 0.0001x^2^–0.0238x +773.87	r = 0.545, P > 0.05
Sucrose	0.25	y = 9E-05 x^3^–0.034x^2^–4.5102x +534.21	r = 0.998, P < 0.01
Honey	0.25	y = 0.0001x^3^–0.0468x^2^ + 5.788x + 535.72	r = 0.995, P < 0.01
Sucrose	0.5	y = 0.0001x^3^–0.0515x^2^ + 6.8273x +405.66	r = 0.998, P < 0.01
Honey	0.5	y = 0.0002x^3^–0.0633x^2^ + 7.8479x + 423.8	r = 0.997, P < 0.01
Sucrose	1.0	y = 0.0002x^3^–0.08x^2^ + 9.9101x + 327.58	r = 0.988, P < 0.01
Honey	1.0	y = 0.0002x^3^–0.0816x^2^ + 10.024x + 406.8	r = 0.994, P < 0.01
Sucrose	1.5	y = 0.0005x^3^–0.1568x^2^ + 15.173x + 433.7	r = 0.952, P < 0.01
Honey	1.5	y = 0.0003x^3^–0.1091x^2^ + 11.863x + 310.27	r = 0.986, P < 0.01
Sucrose	2.0	y = 0.0003x^3^–0.0871x^2^ + 7.6963x + 459.36	r = 0.915, P < 0.01
Honey	2.0	y = 0.0003x^3^–0.0837x^2^ + 8.946x + 318.44	r = 0.980, P < 0.01

### Experiment 2: IVM of bovine oocytes (GV-stage) vitrified in honey and sucrose solutions

**Fig 4:**
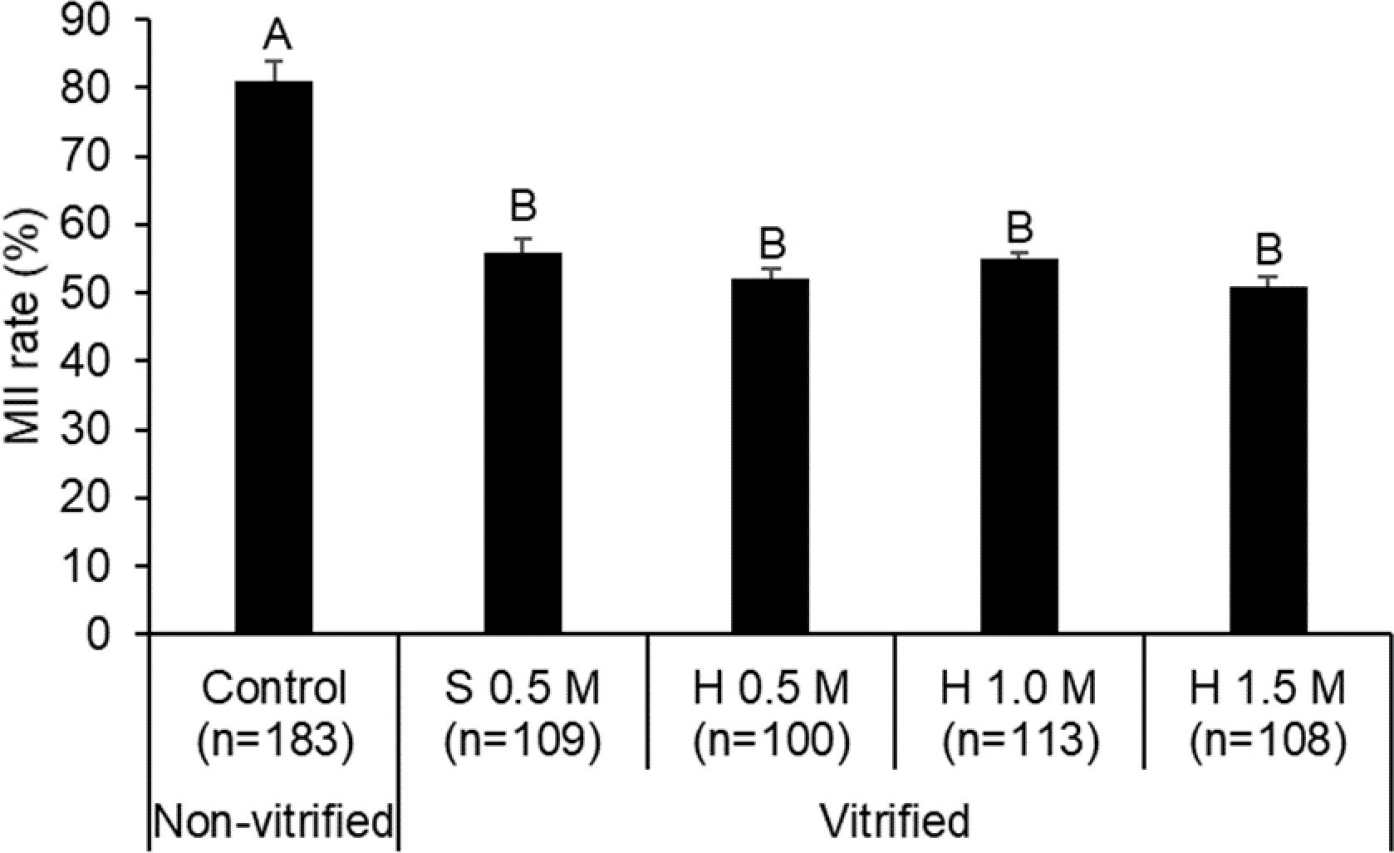
In vitro maturation of bovine oocytes (GV-stage) vitrified in 0.5 M sucrose and different concentrations of honey solutions. Each bar represents mean ± SEM from 5 replicates. Different letters on bars represent differences among treatment groups (P < 0.05). Letter n represents the number of COCs used in control and vitrified groups; MII, metaphase-II.

IVM ability of nonvitrified (control) and vitrified COCs in 0.5 M sucrose or different concentrations of honey was determined. The number of mature oocytes (MII-stage) was greater in the control group than in the vitrified groups (P < 0.05; Fig 4). On the other hand, no significant differences were found in nuclear maturation (MII) rates among honey and sucrose vitrified groups.

### Experiment 3: IVF and IVC of bovine oocytes (GV-stage) vitrified in honey and sucrose solutions

**Fig 5:**
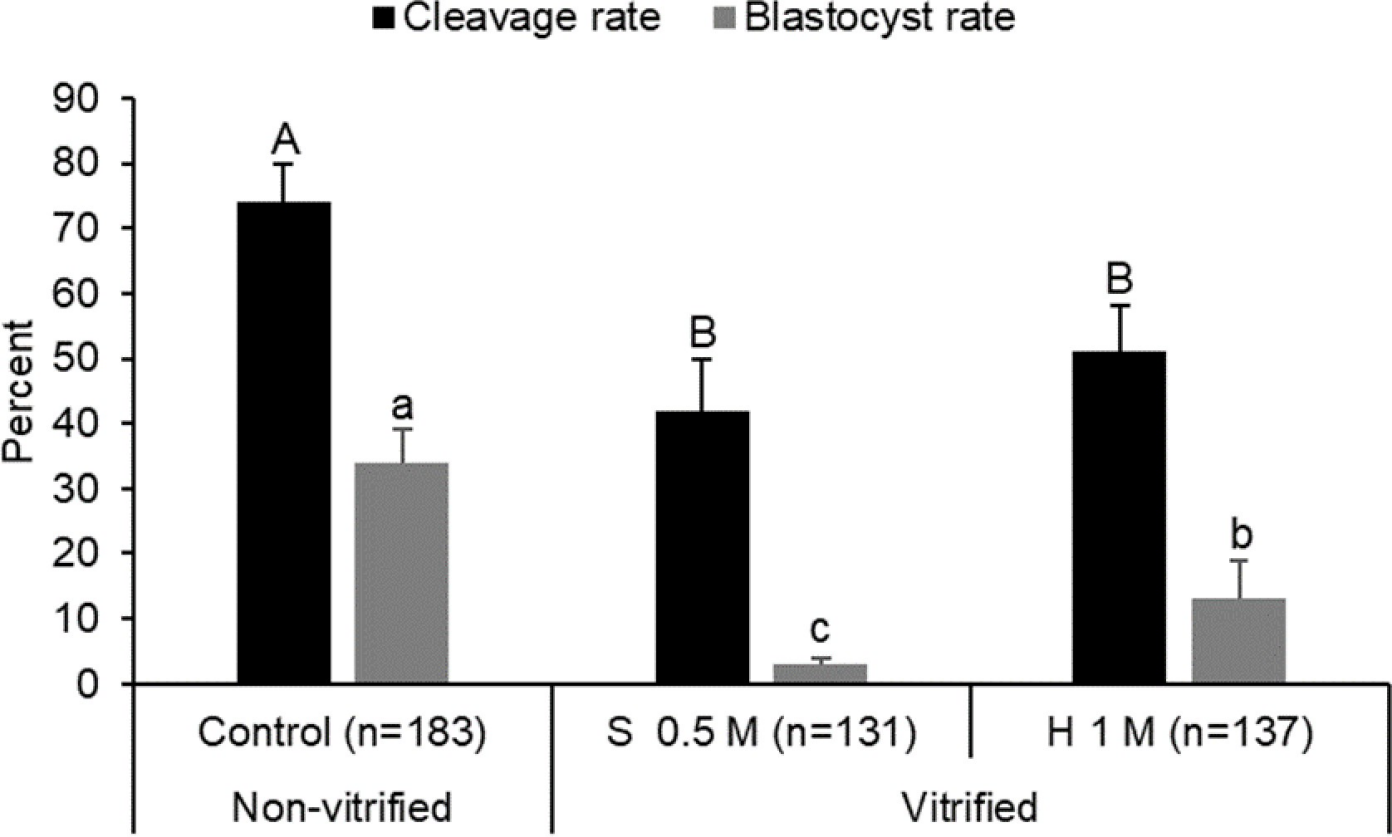
In vitro cleavage and blastocyst rates in oocytes (GV-stage) vitrified in 0.5 M sucrose or 1 M honey solution. Each bar represents mean ± SEM from 5 replicates. Within an embryonic stage, different letters on bars represent difference among treatment groups (P < 0.05). Letter n represents the number of COCs used in control and vitrified groups.

Ooplasm shrinkage between 0.5 sucrose and 1 M honey, used in this experiment, was statistically similar (P > 0.05). Cleavage rates were significantly greater (P < 0.05) in the control group than in vitrified groups (Fig 5). However, cleavage rates between vitrified groups were not significantly different. Blastocyst rate was greater in the nonvitrified (control) group than in vitrified groups, and in honey than sucrose group (P < 0.05; Fig 5).

## Discussion

This is a first report on the use of honey as a nonpermeating CP using a bovine oocyte model. In this study, the volumetric change observed in response to honey or sucrose solutions was virtually in the ooplasm, not in oocyte per se. The volumetric change in ooplasm in honey and sucrose containing solution was concentration-dependent. An inverse relationship was found between ooplasm volume change and concentration of honey or sucrose. Volumetric changes in ooplasm during dehydration in honey- and sucrose-based solutions were similar. The ooplasm regained their approximate original volume during rehydration in 60 s indicating their integrity and viability after exposure to honey or sucrose solutions. This study also demonstrated that COCs (GV-stage) vitrified in a solution containing honey or sucrose had similar maturation and cleavage rates. However, the ability of vitrified-warmed oocytes to reach blastocyst stage in the honey group was significantly greater than in the sucrose group. Thus, honey can be used as a nonpermeating CP in vitrification of bovine oocytes.

Natural honey is a mixture of about 25 saccharides (mainly fructose and glucose), which accounts for approximately 95% dry matter [16]. Fructose and glucose are good osmotic buffers in cryoprotective solutions because they have a lower viscosity than disaccharides [9]. The significant linear relationship between molal concentration of honey and osmotic pressure in honey and sucrose solutions revealed that sugars and other molecules present in honey contribute towards the osmolality of the solution. The ultimate aim of this study was to improve the embryonic development in vitrified GV-stage oocytes. However, in the first experiment, MII oocytes were used as proof of concept of the dehydration ability of honey. Cumulus cells surrounding MII oocytes can be easily removed to monitor volume changes in the ooplasm.

The appropriate concentration of honey and sucrose to be used in vitrification solutions should be based on the ooplasm volume with maximum dehydration without causing any physico-chemical damage to oocytes. A lethal issue for living cells’ dehydration is the loss of bound-water which forms a “hydration shell” around various cellular molecules such as proteins, DNA, RNA and membrane phospholipids, and thus protects their structures and functions [35]. Therefore, during dehydration of oocytes, a delicate balance must be maintained for drawing out free water (which tends to form ice crystals rapidly) while not disturbing the bound water in order to acquire a safe dehydration of the intracellular compartment. Otherwise, the post-warm cell viability will be affected as a result of losing the structural support to intracellular proteins and lipids. According to previous studies, the osmotically inactive volume (*i*.*e*. proportion of cell volume that has no response to extracellular hyperosmotic pressure) of MII bovine oocytes in the presence of NaCl [36] or sucrose [37] were determined to be 24.7 and 26.1% of the isosmotic cell volume, respectively. The osmotically inactive volume of a mature human oocyte is about 19%, indicating that volume of intracellular free-water is approximately 80% of the oocyte volume [38]. The osmotic tolerance limit is about 57% of the original volume to dehydrate human oocytes safely using 0.4 M sucrose [39]. In the present study, the osmotic tolerance threshold of oocyte was set at 50%. The ooplasm shrinkage in 0.5 M sucrose and 1 M honey at 60 s during dehydration was not different (58 vs. 52%, respectively). It was anticipated that 1 M honey would provide similar dehydration but add more nutritive benefits, compared to 0.5 M sucrose. Therefore, cleavage and blastocyst rates of oocytes vitrified in 0.5 M sucrose and 1 M honey were compared. Increasing the extracellular concentrations of nonpermeating CPs in vitrification solution significantly reduced the concentrations of permeating CPs required for intracellular vitrification [3]. Therefore, an ideal vitrification solution should have an appropriate sugar concentration in order to enhance cell dehydration, minimizing the quantity of intracellular permeating CPs while not exceeding the osmotic-tolerance limit of oocytes [3]. Sugar alleviates the requirement for high concentrations of permeating CPs, and thus decreases their toxicity [35, 38].

Polynomial regression analysis indicated a lack of relationship in ooplasm volume change as a function of time in the control group and clearly demonstrated the validity of our procedure to measure the ooplasm volume. However, the ooplasm volume changed over time in all concentrations of sucrose and honey, following third degree cubic regression. The decrease and increase of ooplasm volume during dehydration and rehydration respectively changed linearly during first 30–60 s and then became stable afterwards.

So far, no tangible improvement has been made in oocyte vitrification in spite of using additives in vitrification solution, culture media or by structural modifications. Recently, cAMP modulator forskolin or IBMX in the medium and partial removal of cumulus cells from GV oocytes improved blastocyst development following vitrification [27, 29]. However, these findings must be confirmed. The present study indicated similarities in short term post-warm viability (maturation and cleavage rates) between honey and sucrose groups. However, vitrified-warmed oocytes in the honey group demonstrated better blastocyst development than in the sucrose group (13 vs. 3%) but it was still low. Inadequate cell dehydration leads to formation of large intracellular ice crystals, which can be lethal to cells [40]. Therefore, COCs dehydrated by 1 M honey-based solution had minimal chance of oocyte damage by intracellular ice crystallization during cooling and warming procedures. In the present study, the 0.5 M sucrose group was considered the gold standard since it is the most commonly used sugar in vitrification solution. Our results showed that maturation, cleavage and blastocyst formation rates of the 0.5 M sucrose groups were comparable to other studies on vitrification of bovine GV oocyte using the Cryotop and open pulled straw methods [28, 41]. These results indicate low relationship between cleavage and embryonic (blastocyst) development. In a recent cytotoxicity study in oocytes, the cleavage rate was not considered a reliable parameter for evaluating the oocytes’ viability because toxic effects are reflected in later stages of embryonic development, *i*.*e*. blastocyst than the early cleavage stage [42]. We considered blastocyst formation as a satisfactory parameter for evaluation of post-warm viability of vitrified oocytes.

From another point of view, along with predominant saccharides, natural honey contains number of bioactive substances such as organic acids, enzymes, antioxidants and vitamins. Such a unique composition provides numerous nutritional, biological and pharmacological effects in living cells [16, 17, 19–21, 43, 44]. Therefore, we can speculate that the wide variety of bioactive substances in natural honey might be responsible for better post-warm embryonic development. Honey is composed of a wide variety of amino acids [17, 44, 45], and glycine and alanine help the cell membrane from freezing by stabilizing phospholipids [46]. Recent studies stated that amino acids have been successfully used as nonpermeating CPs for mammalian cells including sperm and oocytes [47–49]. It was demonstrated that addition of glutamine, one of the amino acids in honey, to vitrification solution improved the maturation ability of vitrified-warmed immature bovine oocytes [49].

In conclusion, natural honey 1 M (21.7% w/v) was found to be a suitable nonpermeating CP to dehydrate bovine oocytes sufficiently, and can be used in vitrification solution. Vitrification of oocytes in solution containing 1 M natural honey improved post-warm oocyte viability and embryonic development.

## Supporting information

S1 File (XLS)
